# Symptom burden and palliative care in patients with hematologic malignancies: a single-center experience

**DOI:** 10.1007/s00520-026-10429-z

**Published:** 2026-02-24

**Authors:** Annasofia Holopainen, Taru Kuittinen, Kristiina Tyynelä-Korhonen, Annamarja Lamminmäki

**Affiliations:** 1 Department of Hematology, Kuopio University Hospital, The Wellbeing Services County of North Savo, Kuopio, Finland; 2 University of Eastern Finland, Kuopio , Finland; 3 Palliative Care Center, Päijät-Häme Wellbeing Services County, Lahti, Finland; 4 Cancer Center, Kuopio University Hospital, The Wellbeing Services County of North Savo, Kuopio, Finland

**Keywords:** Hematologic neoplasms, Symptoms, Hospitalization, Terminal care, Palliative medicine

## Abstract

**Background:**

Patients with hematological malignancies are a heterogeneous group with special palliative care needs. They are known to have many symptoms and frequent emergency room visits and hospitalizations in the end-of-life phase. However, there are limited data about the symptom burden and health care service utilization in different diagnostic groups of hematological malignancies.

**Objectives:**

The purpose of this study was to define the symptom burden and characteristics of the palliative care phase in patients with hematological malignancies in different diagnostic groups.

**Methods:**

This retrospective study comprised 195 patients with hematological malignancies who were treated in the Kuopio University Hospital palliative care unit and died between 1.1.2015 and 31.12.2023. Patients were divided into four patient groups: myeloma, lymphoma, leukemia and myelodysplastic syndrome or myeloproliferative neoplasm (MDS/MPN).

**Results:**

All patient groups had a considerable symptom burden, with many emergency room visits and hospital admissions near death. Furthermore, palliative care involvement occurred late. Fatigue, pain, and dyspnea were the most common symptoms in myeloma and lymphoma patients; fatigue, pain and loss of appetite in leukemia patients and fatigue, dyspnea and depression or anxiety in MDS/MPN patients in palliative care.

**Conclusion:**

Patients with hematological malignancies have a significant symptom burden and remarkable palliative care needs at the end of life. More research and awareness of the benefits of palliative care are needed.

## Relevance statement

1. What is already known about the topic?
  Patients with hematological malignancies have a high level of symptoms at the end of life, but they receive less palliative care and at a later stage than other cancer patients. There are insufficient palliative care integration models for hematologic patients, and limited data exist on symptom burden across different diagnostic groups of patients with hematological malignancies.
2. What this paper adds?
  This study demonstrates that patients with hematological malignancies experience a variety of symptoms at the end of life and often require emergency visits and hospitalizations near death. It also provides new information about symptom burden and end-of-life care across different diagnostic groups of hematological malignancies.
3. Implications for practice, theory, or policy?
  Earlier palliative care involvement for patients with hematological malignancies is recommended, along with enhanced collaboration between hematologists and palliative care units.

## Introduction

Palliative care is an active and comprehensive treatment that should be involved in the treatment of all patients facing life-threatening or terminal illnesses. It aims to relieve symptoms and enhance the patient’s quality of life [1]. Integrated palliative care refers to the initiation of palliative care during curative or life-prolonging treatment [2–4].

Hematologic malignancies (HM) represent a heterogeneous group of different diseases with distinct characteristics compared to solid tumors (ST) [2, 5, 6]. Certain hematologic diseases progress rapidly, and there might still be a curative intention even in relapsed settings. Conversely, many of these diseases are known already at diagnosis to be incurable, but patients may live years with the condition [2, 5]. Patients with HM frequently have cytopenias and require blood products and antibiotics, which create special treatment needs [2, 7].

Patients with HM have been described to have symptoms similar to patients with ST, but usually worse performance status near death and even higher symptom burden than other cancer patients [4, 8, 9]. However, patients with HM tend to receive palliative care at a later stage than patients with ST and are more likely to receive chemotherapy or targeted therapy within the last month of life, with prolongation of life often being the main treatment goal [4, 8–12]. Furthermore, these patients have been shown to have many emergency room (ER) visits and hospitalizations at the end-of-life (EOL) phase [4, 8–12].

Palliative care involvement for patients with HM remains insufficient and is frequently delayed, underscoring the urgent need for further research [3, 4, 10, 12, 13]. Moreover, data regarding symptom burden across different diagnostic groups of HM are limited. Therefore, we performed this retrospective study to investigate the symptoms experienced by patients with HM in various diagnostic groups and to evaluate how these patients were treated during the palliative care phase.

## Materials and methods

### Study design and patients

Kuopio University Hospital (KUH) is one of the five university hospitals in Finland. The study cohort included all 195 adult patients with hematological malignancies who had a documented palliative care decision, were treated in the KUH palliative care unit, and died between 1.1.2015 and 31.12.2023. 

Palliative care decision was defined as either the discontinuation of anticancer therapy or the determination not to initiate it in cases where hematological malignancy was progressive or refractory, and no suitable therapeutic options remained, or where the patient was too frail to undergo treatment. Following this decision, the therapeutic objective shifted from curative or life-prolonging intent to symptom management and end-of-life care planning. At KUH, clinical decisions were made either by the responsible physician or through multidisciplinary discussions among hematologists and oncologists, after which patients were referred to the palliative care unit.

Included patients were divided into four diagnostic groups: multiple myeloma (*n* = 34), lymphoma (*n* = 68; all subtypes, but the majority had non-Hodgkin’s lymphoma), leukemia (*n* = 61; 49 had de novo or secondary acute myeloid leukemia, five acute lymphoblastic leukemia, and seven chronic myeloid or lymphocytic leukemia), and myelodysplastic (MDS) or myeloproliferative (MPN) disease (*n* = 32; four with MPN, the others MDS). Due to the small number of patients, no statistical comparisons between the diagnostic groups were performed.

### Health care services in Finland

Primary health care is provided by a municipal health center, while secondary care is delivered through 20 district hospitals and tertiary care at five university hospitals. Palliative care services are categorized into general and specialist levels and are provided in primary, secondary, and tertiary levels of the health care system.

Finnish Institute for Health and Welfare defines primary health care as the publicly organized, first level of health services provided through health and social services centers. It includes preventive care, diagnosis, treatment, rehabilitation, and coordination with specialized care, and is universally available to all residents. Primary health care wards are inpatient units in health care centers that provide care for patients who do not require specialized hospital treatment. These wards deliver short-term inpatient treatment, rehabilitation, and EOL care.

### Data collection

This study was approved by the local ethics committee in January 2024. All data were collected by the author from medical records in May 2024.

Patient data were collected retrospectively from medical records and entered into the SPSS program (version 29.0.1.0) and analyzed. Collected data included patients’ characteristics (age, sex, marital status, and co-morbidities), diagnosis, disease-specific information, previous medical history and previous anticancer treatment, symptoms, medications and treatments during palliative care, palliative and DNR (do not resuscitate) decision dates, ER visits and hospitalizations during the last month of life and the time and place of death.

Information on patients’ symptoms was collected from the medical records of the first contact and the last contact in KUH palliative care unit. The Edmonton Symptom Assessment Scale (ESAS) was systematically utilized during the study period. Symptom data were collected from each patient at the beginning of every visit by a nurse using the ESAS. Due to the retrospective nature of the study, all detailed information from the ESAS was not always documented in patient charts. The presence of a symptom was coded “yes” if it was mentioned in the medical records and “no” if it was not mentioned or if the patient did not experience that symptom. We included information about the following symptoms: pain (at rest or in motion), fatigue, dyspnea, loss of appetite, nausea, constipation, fever, insomnia, depression, or anxiety.

Altogether 14 of all patients were transferred to other hospital districts, and thus data regarding the last 30 days was incomplete (Table 2). Nevertheless, the date of death was available for all patients.

## Results

### Patient characteristics

Table 1 presents the characteristics of the 195 patients included in the study, of whom 17.4% (*n* = 34) had multiple myeloma. Their mean age at death was 72.9 years and 58.8% were male. Patients with lymphoma comprised 34.9% (*n* = 68) of all patients. Their mean age at death was 74.8 years, and 63.2% were male. One third (31.3%) (n = 61) of all patients had leukemia, their mean age at death was 70.4 years, and 54.1% were male. Moreover, 16.4% (*n* = 32) of all patients had MDS or MPN, their mean age at death was 76.6 years, and 53.1% were male. Most patients had comorbidities, as shown in Table 1. The most common comorbidity was cardiovascular disease in all patient groups. Patients with lymphoma and MDS/MPN had the most comorbidities.

**Table 1:** Patient characteristics (*n* = 195)

	Myeloma (***n*** = 34)		Lymphoma (***n*** = 68)		Leukemia (***n*** = 61)		MDS/MPN (***n*** = 32)	
	Mean/*N*	SD/%	Mean/*N*	SD/%	Mean/*N*	SD/%	Mean/*N*	SD/%
Age at death (years)	72.9	1.7	74.8	1.7	70.4	1.6	76.6	1.8
Sex								
Male	20	58.8%	43	63.2%	33	54.1%	17	53.1%
Female	14	41.2%	25	36.8%	28	45.9%	15	46.9%
Marital status								
Married	24	70.6%	44	64.7%	36	59.0%	18	56.3%
Not married	10	29.4%	24	35.3%	25	41.0%	14	43.7%
Number of comorbidities								
0–1	14	41.2%	17	25.0%	9	14.8%	4	12.5%
2–4	11	32.4%	31	45.6%	39	63.9%	19	59.4%
5 or more	9	26.5%	20	29.4%	13	21.3%	9	28.1%
Comorbidities								
Cardiovascular	13	38.2%	43	63.2%	25	41.0%	18	56.3%
Pulmonar	3	8.8%	13	19.1%	8	13.1%	9	28.1%
Dementia	5	14.7%	15	22.1%	7	11.5%	3	9.4%

*n* number, *MDS/MPN* myelodysplastic syndrome or myeloproliferative neoplasm

### Characteristics of palliative care

The characteristics of palliative care are shown in Table 2. The median time from the last treatment to death was 51 days among patients with myeloma, 148 days among patients with lymphoma, 120 days among patients with leukemia, and 213 days among patients with MSD/MPN. Furthermore, the median time from the palliative care decision to death was 40 days among patients with myeloma, 67 days among patients with lymphoma, 48 days among patients with leukemia, and 126 days among patients with MSD/MPN.

**Table 2:** Characteristics of palliative care

	Myeloma (***n*** = 34)		Lymphoma (***n*** = 68)		Leukemia (***n*** = 61)		MDS/MPN (***n*** = 32)	
	Median/*N*	(5th-95th percentiles)/%	Median/*N*	(5th-95th percentiles)/%	Median/*N*	(5th-95th percentiles)/%	Median/*N*	(5th-95th percentiles)/%
Time from last treatment to death (days)	51	(11–1266)	148	(11–1262)	120	(20–564)	213	(57–714)
Time from palliative care decision to death (days)	40	(4–1116)	67	(3–843)	48	(3–281)	126	(20–602)
Time from DNR to death (days)	66	(7–1194)	60	(5–1094)	57	(3–517)	173	(17–766)
Palliative radiotherapy used								
Yes	12	35.3%	33	48.5%	2	3.3%	0	0.0%
No	22	64.7%	35	51.5%	59	96.7%	32	100%
ER visit within last 30 days								
Yes	23	67.6%	31	45.6%	34	55.7%	11	34.4%
No	11	32.4%	36	52.9%	23	37.7%	20	62.5%
Hospitalization within last 30 days								
Yes	21	61.8%	26	38.2%	32	54.2%	8	25.0%
No	13	38.2%	41	60.3%	23	39.0%	23	71.9%
PCA in use before death								
Yes	12	35.3%	23	33.8%	16	26.2%	8	25.0%
No	22	64.7%	44	64.7%	40	65.6%	22	68.8%
Place of death								
Hospital	2	5.9%	8	11.8%	5	8.2%	0	0.0%
Hospice	9	26.5%	18	26.5%	15	24.6%	13	40.6%
Primary health care ward	18	52.9%	32	47.1%	28	45.9%	13	40.6%
Home	3	8.8%	5	7.3%	8	13.1%	4	12.5%
Other/Unknown	2	5.9%	5	7.3%	5	8.2%	2	6.3%

*n *number, *MDS/MPN* myelodysplastic syndrome or myeloproliferative neoplasm, *DNR* do not resuscitate, *ER* emergency room, *PCA* patient-controlled analgesia

Palliative radiotherapy was given to 35.3% of patients with myeloma and 48.5% of patients with lymphoma. None of the patients with MDS/MPN, and 3.3% of patients with leukemia had palliative radiotherapy.

Within the last 30 days of life. the majority (67.6%) of patients with myeloma had one or more ER visits and 61.8% were hospitalized at least once. Patients with MDS/MPN had the fewest ER visits (34.4%) of all diagnostic groups, and 25.0% were hospitalized in the last 30 days of life. The most common reasons for an ER visit among patients with myeloma was nonspecific complaints (NSC) (30.4%) and fever or infection (21.7%). Among patients with lymphoma and leukemia the most common reason was fever due to infection (32.3%/57.6%) and NSC (22.6%/12.1%). Anemia or the need for transfusion was the most common reason among patients with MDS/MPN (27.3%).

Most patients in our dataset died in hospice or in primary care. Thus, 26.5% of patients with myeloma and lymphoma and 24.6% of patients with leukemia died in hospice, while 52.9% of patients with myeloma, 47.1% with lymphoma and 45.9% with leukemia died in primary health care wards. Patients with lymphoma had the highest likelihood of dying in hospital (11.8%). None of the patients with MDS/MPN died in the hospital; most died either in hospice (40.6%) or in primary health care wards (40.6%).

### Symptom burden in palliative care

**Fig. 1:**
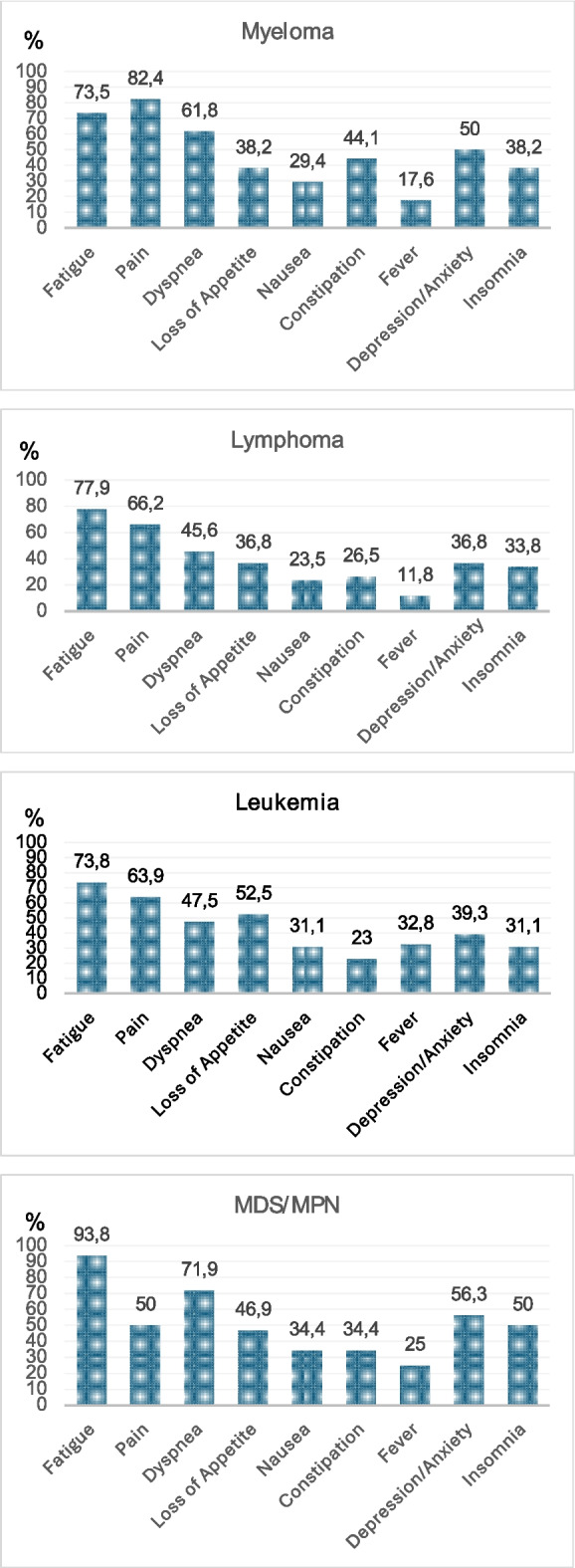
Frequency of selected symptoms in different diagnostic groups. MDS/MPN = myelodysplastic syndrome or myeloproliferative neoplasm

The frequency of symptoms in different patient groups is presented in Fig. 1. Fatigue was reported by most patients: 73.5% with myeloma, 77.9% with lymphoma, 73.8% with leukemia, and 93.8% with MDS/MPN. Pain was experienced by 82.4% of patients with myeloma, 66.2% with lymphoma, 63.9% with leukemia, and 50.0% with MDS/MPN. The majority of patients with MDS/MPN (71.9%) and over half (61.8%) of patients with myeloma experienced dyspnea. Moreover, 47.5% of patients with leukemia and 45.6% of patients with lymphoma reported dyspnea. Fever was a quite common symptom among patients with leukemia, of whom 32.8% had episodes of fever. Furthermore, 25.0% of patients with MDS/MPN, 17.6% of patients with myeloma, and 11.8% of patients with lymphoma had fever during palliative care. Depression and anxiety affected 50.0% of patients with myeloma, 36.8% of patients with lymphoma, 39.3% of patients with leukemia, and 56.3% of patients with MDS/MPN.

### Medications in palliative care: opioids and corticosteroids

During palliative care 85.3% of patients with myeloma regularly used opioids, and 91.2% used breakthrough opioids for pain. Moreover, 74.6% of patients with lymphoma, 53.3% of patients with leukemia, and 56.3% of patients with MDS/MPN regularly used opioids, and 97.1% of patients with lymphoma, 93.4% of patients with leukemia, and 87.5% of patients with MDS/MPN used breakthrough opioids.

Corticosteroids were frequently used in palliative care in all diagnostic groups. Among patients with myeloma, 58.8% received corticosteroids, with 40% for symptom control and 35% for the management of nausea, loss of appetite or fatigue. Use of corticosteroids was common among patients with lymphoma (86.8%). Half (55%) used them for symptom control, 15% for fever and 13.3% for nausea, loss of appetite or fatigue. Moreover, 55.7% of patients with leukemia used corticosteroids during palliative care, 46.4% for fever and 17.9% for nausea, loss of appetite or fatigue. Half (50%) of the patients with MSD/MPN used corticosteroids, 44% for fever control and 38% for nausea, loss of appetite or fatigue.

The most used corticosteroid was prednisolone in all diagnostic groups. The average dose varied from 10 to 60 mg daily. Dexamethasone was also used, with the average dose varying from 2 to 6 mg daily.

## Discussion

In this study fatigue, pain and dyspnea were the most common symptoms among patients with myeloma and lymphoma; fatigue, pain and loss of appetite in patients with leukemia and fatigue, dyspnea and depression/anxiety in patients with MDS/MPN. Furthermore, all the patient groups had ER visits and hospital admissions near death, and palliative care involvement occurred late in the disease trajectory. According to WHO, palliative care should be initiated early in the disease trajectory, not just in the final stages [1]. This emphasizes the importance of timely recognition of palliative care needs and fostering co-operation between hematologists and palliative care services.

Notably, in our cohort, all patients received palliative care, and hospital deaths seemed to be less frequent and hospice referrals more common compared to previous studies investigating EOL care among patients with HM [14–19]. Taken together, these findings may suggest that palliative care involvement increases the likelihood of patients dying outside the hospital, particularly in hospice, compared with those without such involvement.

Patients with myeloma suffer from various symptoms [10, 20–22]. Pain is the most frequent symptom and is usually present during the whole course of the disease, strongly affecting quality of life [7, 23]. Our study supported this observation, as pain was the most common symptom, affecting most patients with myeloma. Additionally, patients in our cohort experienced fatigue, dyspnea, constipation, loss of appetite, and psychological symptoms. In previous studies, frequently reported symptoms were fatigue, peripheral neuropathy, constipation, depression, insomnia, loss of appetite, and dyspnea [22, 23].

Although overall survival for patients with myeloma has improved in recent years, the disease remains incurable [7]. According to our results, patients with myeloma seemed to receive anticancer treatment closer to death, and palliative care involvement occurred later than in patients from other diagnostic groups. These patients also had more ER visits and were hospitalized more frequently during the last month of life than those in other groups. Previous studies have reported similar findings, indicating that patients with myeloma often have delayed involvement in palliative care [10, 21]. However, palliative care for patients with myeloma has been shown to improve quality of life and symptom management [7, 24]. Furthermore, a study by Porta-sales et al. [23] demonstrated the benefits and feasibility of early palliative care for patients with myeloma, with effective control of pain and other symptoms. Earlier palliative care involvement has been associated with reduced use of aggressive care and fewer hospitalizations at EOL, both in patients with myeloma and in those with other malignancies [24–26].

Incidence of lymphoma is increasing, encompassing diverse subtypes, treatment regimens, and outcomes, among which non-Hodgkin lymphoma (NHL) is the most common [27]. In our study, the three most frequently reported symptoms in patients with lymphoma were fatigue, pain, and dyspnea. For comparison, Jin et al. [28] reported that the most common symptoms in patients with aggressive NHL were disturbed sleep, fatigue, difficulty remembering, dry mouth, and distress.

In our study, most patients with lymphoma were older and diagnosed with NHL. Although the median time from the last treatment to death in this group was five months, palliative care involvement occurred approximately two months before death. Nearly half of the patients had ER visits, and over one-third were hospitalized during the last month of life, and patients with lymphoma appeared to have the highest likelihood of dying in hospital. Similarly, Johnson et al. [14] reported high health care utilization and low hospice use among older adults with aggressive NHL at EOL, with 70% hospitalized and more than half dying in a hospital or health care facility. However, palliative care consultation was associated with a greater likelihood of hospice utilization.

Acute leukemias can progress rapidly, are associated with high morbidity, and often have poor prognosis, particularly in older patients [15, 29–31]. In our study, the most common symptoms among patients with leukemia were fatigue, pain, dyspnea, loss of appetite, and depression or anxiety. Moreover, one-third of the patients experienced episodes of fever, potentially due to infection or as a symptom of progressive leukemia. More than half of the patients had ER visits and were hospitalized in the last month of life.

In a study by Zimmermann et al. [15], low and late involvement of palliative care among patients with acute leukemia was reported, with only six percent of patients who died receiving palliative care, a mean of six days between referral and death, and none being referred to psychiatric or psychological services. Conversely, the benefits of integrated palliative care for patients with acute myeloid leukemia (AML) were demonstrated in a study by El-Jawahri et al. [3], which showed improvements in patient-reported quality of life and psychological outcomes, as well as increased EOL care discussions and reduced chemotherapy use near death. Similar results were reported by Potenza et al. [32], who observed better palliative care quality indicators and less aggressive EOL treatment with early palliative care among patients with AML.

Myelodysplastic syndromes and myeloproliferative neoplasms (MDS/MPN) are a heterogeneous group of clonal stem cell disorders with variable disease trajectories and prognoses, sharing a tendency to progress to acute leukemia over time [31, 33, 34]. Patients with MDS often experience significant quality of life impairment and co-morbidities, yet they are frequently excluded from studies on symptoms and EOL phase in patients with HM [33]. In our study, patients with MDS/MPN were the oldest and had the highest burden of comorbidities. Fatigue was the most prevalent symptom, reported by over 90% of patients. Consistently, fatigue is known to be a highly prevalent symptom among patients with MDS and appears to correlate only weakly with the degree of anemia [35]. Other commonly observed symptoms in our cohort included dyspnea, depression or anxiety, pain, and insomnia. Furthermore, one-quarter of patients experienced episodes of fever, which may have been related to infections or disease progression.

Patients with MDS/MPN seemed to have the longest intervals from last treatment, palliative care involvement, and DNR decision to death, which may reflect both the heterogeneity of the disease and the fact that treatment in elderly patients is often palliative from the time of diagnosis. Interestingly, these patients were less frequently hospitalized, had fewer ER visits during the last month of life, and none of them died in hospital. These findings might suggest that timely involvement of palliative care may help reduce health care service utilization near death and guide patients toward an appropriate place of death.

Early integration of palliative care is essential for all patients with hematological malignancies. Together with advance care planning and timely hospice referral, it may reduce delays in care and decrease the frequency of ER visits and hospitalizations near death [3, 30–32, 36, 37]. Furthermore, early palliative care interventions can enhance symptom management, provide psychological support, and assist both patients and their families in coping with an incurable disease [2, 3, 13]. Nevertheless, models for the early integration of palliative care among patients with HM remain limited in Finland, underscoring the urgent need for further research and development in this area.

### Strengths and limitations

To the best of our knowledge, this is the first study to examine symptom burden and EOL characteristics in patients with HM in Finland. All data were collected by one person (A.H.). The study included 195 patients comprising different hematological diagnostic groups. It provides a comprehensive overview of the symptom burden and palliative care needs across diagnostic groups and identifies areas for potential development in palliative care for patients with HM.

This was a retrospective study with inherent limitations. Systematic data analysis was not possible in the EOL phase. Records of patients’ symptoms (ESAS questionnaire) were not always documented in detail. Also, some data were missing or not recorded regarding patients dying in other hospital districts. Due to the small number of patients, no statistical comparisons between the diagnostic groups were performed. Furthermore, as this was a single-center study, the generalizability of the findings is limited.

## Conclusions

The results of this retrospective study demonstrate that patients with hematological malignancies experience a significant physical and psychological symptom burden and high health care utilization at EOL. Palliative care involvement is often delayed, highlighting the importance of increasing awareness among hematologists, who play a crucial role in enabling earlier referral to palliative care.

## Data Availability

No datasets were generated or analysed during the current study.

## References

[1] WHO (2020) Global Atlas of Palliative Care, 2nd Edition, London.

[2] Shaulov A, Aviv A, Alcalde J, Zimmermann C (2022) Early integration of palliative care for patients with haematological malignancies. Br J Haematol 199:14–30. 10.1111/bjh.18286 35670630 10.1111/bjh.18286 PMC9796711

[3] El-Jawahri A, LeBlanc TW, Kavanaugh A et al (2021) Effectiveness of integrated palliative and oncology care for patients with acute myeloid leukemia: a randomized clinical trial. JAMA Oncol 7:238–245. 10.1001/jamaoncol.2020.6343 33331857 10.1001/jamaoncol.2020.6343 PMC7747042

[4] Oechsle K (2019) Palliative care in patients with hematological malignancies. Oncol Res Treat 42:25–30. 10.1159/000495424 30537761 10.1159/000495424

[5] El-Jawahri A, Nelson AM, Gray TF et al (2020) Palliative and end-of-life care for patients with hematologic malignancies. J Clin Oncol 38:944–953. 10.1200/JCO.18.02386 32023164 10.1200/JCO.18.02386 PMC8462532

[6] Webb JA, Foxwell AM, Jones CA et al (2019) Top ten tips palliative care clinicians should know about caring for patients with hematologic malignancies. J Palliat Med 22:1449–1454. 10.1089/jpm.2019.0332 31329005 10.1089/jpm.2019.0332

[7] Pallotti MC, Rossi R, Scarpi E et al (2022) Patients with multiple myeloma referred for palliative care consultation: from retrospective analysis to future directions to improve clinical outcomes. Support Care Cancer 30:2293–2298. 10.1007/s00520-021-06560-8 34718886 10.1007/s00520-021-06560-8 PMC8795014

[8] Manitta V, Zordan R, Cole-Sinclair M et al (2011) The symptom burden of patients with hematological malignancy: a cross-sectional observational study. J Pain Symptom Manage 42:432–442. 10.1016/j.jpainsymman.2010.12.008 21477979 10.1016/j.jpainsymman.2010.12.008

[9] LeBlanc TW, Smith JM, Currow DC (2015) Symptom burden of haematological malignancies as death approaches in a community palliative care service: a retrospective cohort study of a consecutive case series. Lancet Haematol 2:e334-338. 10.1016/S2352-3026(15)00111-8 26688486 10.1016/S2352-3026(15)00111-8

[10] Hui D, Didwaniya N, Vidal M et al (2014) Quality of end-of-life care in patients with hematologic malignancies: a retrospective cohort study. Cancer 120:1572–1578. 10.1002/cncr.28614 24549743 10.1002/cncr.28614 PMC4013204

[11] Verhoef M-J, de Nijs EJM, Ootjers CS et al (2020) End-of-life trajectories of patients with hematological malignancies and patients with advanced solid tumors visiting the emergency department: the need for a proactive integrated care approach. Am J Hosp Palliat Care 37:692–700. 10.1177/1049909119896533 31867978 10.1177/1049909119896533 PMC7361664

[12] Gebel C, Ditscheid B, Meissner F et al (2024) Utilization and quality of palliative care in patients with hematological and solid cancers: a population-based study. J Cancer Res Clin Oncol 150. 10.1007/s00432-024-05721-6 38607376 10.1007/s00432-024-05721-6 PMC11014814

[13] Moreno-Alonso D, García BG, Madrid-Alejos C et al (2025) Bridging the gap: an updated systematic integrative review of palliative care integration in hematological malignancies. Semin Oncol Nurs 41. 10.1016/j.soncn.2025.151962 40841290 10.1016/j.soncn.2025.151962

[14] Johnson PC, Markovitz NH, Yi A et al (2022) End-of-life care for older adults with aggressive non-Hodgkin lymphoma. J Palliat Med 25:728–733. 10.1089/jpm.2021.0228 34724798 10.1089/jpm.2021.0228 PMC9360173

[15] Zimmermann C, Yuen D, Mischitelle A et al (2013) Symptom burden and supportive care in patients with acute leukemia. Leuk Res 37:731–736. 10.1016/j.leukres.2013.02.009 23490030 10.1016/j.leukres.2013.02.009 PMC3808347

[16] Abbasi S, Roller J, Abdallah A-O et al (2021) Hospitalization at the end of life in patients with multiple myeloma. BMC Cancer 21. 10.1186/s12885-021-08079-x 33789626 10.1186/s12885-021-08079-x PMC8011131

[17] Mohyuddin GR, Sinnarajah A, Gayowsky A et al (2022) Quality of end-of-life care in multiple myeloma: a 13-year analysis of a population-based cohort in Ontario, Canada. Br J Haematol 199:688–695. 10.1111/bjh.18401 35949180 10.1111/bjh.18401

[18] Cheng H-W, Li C-W, Chan K-Y et al (2015) End-of-life characteristics and palliative care provision for elderly patients suffering from acute myeloid leukemia. Support Care Cancer 23:111–116. 10.1007/s00520-014-2333-x 24996833 10.1007/s00520-014-2333-x

[19] Skåreby E, Fürst P, von Bahr L (2025) End-of-life care in hematological malignancies - a nationwide comparative study on the Swedish Register of Palliative Care. PLoS One 20. 10.1371/journal.pone.0312910 40299928 10.1371/journal.pone.0312910 PMC12040083

[20] Johnsen AT, Tholstrup D, Petersen MA et al (2009) Health related quality of life in a nationally representative sample of haematological patients. Eur J Haematol 83:139–148. 10.1111/j.1600-0609.2009.01250.x 19284418 10.1111/j.1600-0609.2009.01250.x PMC2730555

[21] Ebert RPC, Magnus MM, Toro P et al (2023) Hematologic malignancies patients face high symptom burden and are lately referred to palliative consultation: analysis of a single center experience. Am J Hosp Palliat Care 40:761–764. 10.1177/10499091221132285 36205034 10.1177/10499091221132285

[22] Ramsenthaler C, Osborne TR, Gao W et al (2016) The impact of disease-related symptoms and palliative care concerns on health-related quality of life in multiple myeloma: a multi-centre study. BMC Cancer 16. 10.1186/s12885-016-2410-2 27387201 10.1186/s12885-016-2410-2 PMC4937527

[23] Porta-Sales J, Guerrero-Torrelles M, Moreno-Alonso D et al (2017) Is early palliative care feasible in patients with multiple myeloma? J Pain Symptom Manage 54:692–700. 10.1016/j.jpainsymman.2017.04.012 28807703 10.1016/j.jpainsymman.2017.04.012

[24] Giusti D, Colaci E, Pioli V et al (2024) Early palliative care versus usual haematological care in multiple myeloma: retrospective cohort study. BMJ Support Palliat Care 14:291–294. 10.1136/spcare-2023-004524 37751995 10.1136/spcare-2023-004524 PMC11347215

[25] Temel JS, Greer JA, Muzikansky A et al (2010) Early palliative care for patients with metastatic non–small-cell lung cancer. N Engl J Med 363:733–742. 10.1056/NEJMoa1000678 20818875 10.1056/NEJMoa1000678

[26] Zimmermann C, Swami N, Krzyzanowska M et al (2014) Early palliative care for patients with advanced cancer: a cluster-randomised controlled trial. Lancet 383:1721–1730. 10.1016/S0140-6736(13)62416-2 24559581 10.1016/S0140-6736(13)62416-2

[27] Shen Z, Tan Z, Ge L et al (2024) The global burden of lymphoma: estimates from the Global Burden of Disease 2019 study. Public Health 226:199–206. 10.1016/j.puhe.2023.11.023 38086101 10.1016/j.puhe.2023.11.023

[28] Jin J, Ren S, Zhang W et al (2024) Symptom burden and health-related quality of life in patients with aggressive non-Hodgkin lymphoma in China: a cross-sectional study. Blood 144. 10.1182/blood-2024-194707 10.1186/s12885-025-14730-8 PMC12400746 40890641

[29] Rafiq N, Khan MH, Sahibzada M et al (2024) Recent developments and challenges in the treatment of acute leukemia and myelodysplastic syndromes: a systematic review. Cureus 16. 10.7759/cureus.72599 39610611 10.7759/cureus.72599 PMC11604246

[30] Iyer N, Doshi K, Kasire SP, et al (2025) Nationwide trends and disparities in end-of-life care for acute myeloid leukemia: a 2019–2021 NIS Analysis of Palliative Care Utilization and Hospitalization Costs. Am J Hosp Palliat Care 10499091251353759. 10.1177/10499091251353759 10.1177/10499091251353759 40552779

[31] LoCastro M, Sanapala C, Mendler JH et al (2023) Advance care planning in older patients with acute myeloid leukemia and myelodysplastic syndromes. J Geriatr Oncol 14. 10.1016/j.jgo.2022.09.003 36100548 10.1016/j.jgo.2022.09.003 PMC9974785

[32] Potenza L, Scaravaglio M, Fortuna D et al (2021) Early palliative/supportive care in acute myeloid leukaemia allows low aggression end-of-life interventions: observational outpatient study. BMJ Support Palliat Care. 10.1136/bmjspcare-2021-002898 34750145 10.1136/bmjspcare-2021-002898

[33] Nickolich M, El-Jawahri A, LeBlanc TW (2016) Palliative and end-of-life care in myelodysplastic syndromes. Curr Hematol Malig Rep 11:434–440. 10.1007/s11899-016-0352-z 27704467 10.1007/s11899-016-0352-z

[34] Fletcher SA, Cronin AM, Zeidan AM et al (2016) Intensity of end-of-life care for patients with myelodysplastic syndromes: findings from a large national database. Cancer 122:1209–1215. 10.1002/cncr.29913 26914833 10.1002/cncr.29913

[35] Steensma DP, Heptinstall KV, Johnson VM et al (2008) Common troublesome symptoms and their impact on quality of life in patients with myelodysplastic syndromes (MDS): results of a large internet-based survey. Leuk Res 32:691–698. 10.1016/j.leukres.2007.10.015 18054795 10.1016/j.leukres.2007.10.015

[36] Arias-Rojas M, Leiserovich N, Castaño M, Carreño-Moreno S (2025) Experiences of cancer patients in palliative care with advanced care planning: a systematic review and meta-synthesis of qualitative studies. Eur J Oncol Nurs Off J Eur Oncol Nurs Soc 76. 10.1016/j.ejon.2025.102868 10.1016/j.ejon.2025.102868 40179535

[37] Hui D, Bruera E (2020) Models of palliative care delivery for patients with cancer. J Clin Oncol Off J Am Soc Clin Oncol 38:852–865. 10.1200/JCO.18.02123 10.1200/JCO.18.02123 PMC7082156 32023157

